# Congenital myasthenic syndrome with episodic apnoea: clinical, neurophysiological and genetic features in the long-term follow-up of 19 patients

**DOI:** 10.1007/s00415-017-8689-3

**Published:** 2017-11-30

**Authors:** Grace McMacken, Roger G. Whittaker, Teresinha Evangelista, Angela Abicht, Marina Dusl, Hanns Lochmüller

**Affiliations:** 1 John Walton Muscular Dystrophy Research Centre, MRC Centre for Neuromuscular Diseases, Institute of Genetic Medicine, Newcastle University, Newcastle upon Tyne, UK; 2 Institute of Neuroscience, Newcastle University, Newcastle upon Tyne, UK; 3 Friedrich-Baur-Institute, Ludwig Maximilians University, Munich, Germany

**Keywords:** Congenital myasthenic syndrome, Neuromuscular disease, Neurophysiology, Neuromuscular junction

## Abstract

**Background:**

Congenital myasthenic syndrome with episodic apnoea (CMS-EA) is a rare but potentially treatable cause of apparent life-threatening events in infancy. The underlying mechanisms for sudden and recurrent episodes of respiratory arrest in these patients are unclear. Whilst CMS-EA is most commonly caused by mutations in *CHAT*, the list of associated genotypes is expanding.

**Methods:**

We reviewed clinical information from 19 patients with CMS-EA, including patients with mutations in *CHAT*, *SLC5A7* and *RAPSN*, and patients lacking a genetic diagnosis.

**Results:**

Lack of genetic diagnosis was more common in CMS-EA than in CMS without EA (56% *n* = 18, compared to 7% *n* = 97). Most patients manifested intermittent apnoea in the first 4 months of life (74%, *n* = 14). A degree of clinical improvement with medication was observed in most patients (74%, *n* = 14), but the majority of cases also showed a tendency towards complete remission of apnoeic events with age (mean age of resolution 2 years 5 months). Signs of impaired neuromuscular transmission were detected on neurophysiology studies in 79% (*n* = 15) of cases, but in six cases, this was only apparent following specific neurophysiological testing protocols (prolonged high-frequency stimulation).

**Conclusions:**

A relatively large proportion of CMS-EA remains genetically undiagnosed, which suggests the existence of novel causative CMS genes which remain uncharacterised. In light of the potential for recurrent life-threatening apnoeas in early life and the positive response to therapy, early diagnostic consideration of CMS-EA is critical, but without specific neurophysiology tests, it may go overlooked.

## Background

**Fig. 1:**
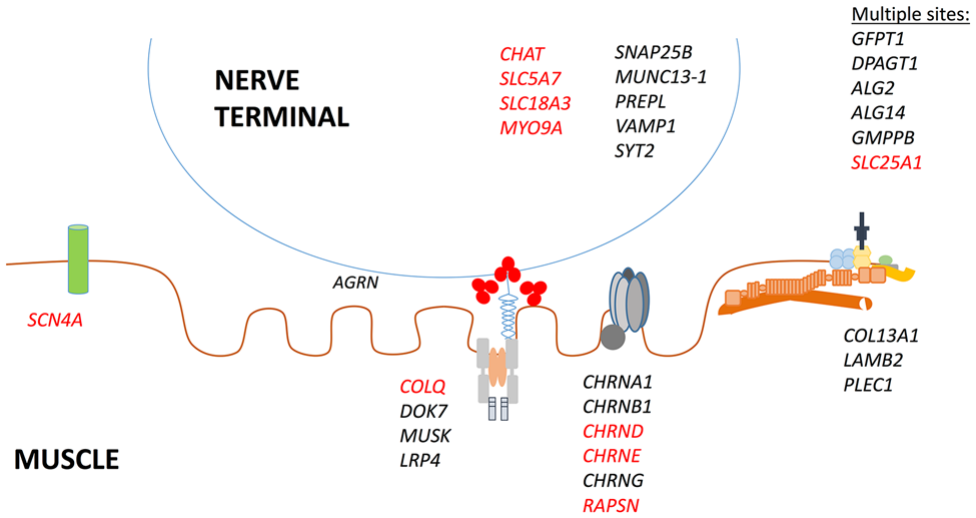
Heterogeneity of genetic defects in CMS: Mutations are described in genes encoding pre-synaptic, synaptic and post-synaptic proteins, proteins of the extracellular matrix and dystrophin-associated glycoprotein complex and in ubiquitously expressed proteins involved in glycosylation (*GFPT1, DPAGT1, ALG2, ALG14, GHMPPB*) and mitochondrial function (*SLC25A1*) which may act at multiple sites. Genes in which mutations have been previously described in patients with EA are highlighted in red

Congenital myasthenic syndromes (CMS) are a group of disorders caused by mutations in genes encoding proteins responsible for the function and integrity of the neuromuscular junction (NMJ), resulting in impairment of the safety margin necessary for reliable neuromuscular transmission. The first identified mutations were in genes encoding subunits of the acetylcholine receptors (AChRs), and these remain the most common subtypes of CMS worldwide today [1]. However, in recent years the discovery of CMS-related genes has accelerated, and to date over 30 genes have been implicated (Fig. 1).

Although the clinical spectrum is increasingly diverse, CMS are characterised by fatigable weakness typically of early onset, a positive family history, and abnormal neurophysiology tests, namely repetitive nerve stimulation (RNS) or single-fibre EMG (SFEMG) [1]. In RNS, supramaximal electrical stimulation is delivered to a motor nerve via surface electrodes. In normal muscle, the resulting response (known as the compound muscle action potential or CMAP) amplitude remains constant over a wide range of frequencies, since the safety margin for neuromuscular transmission is large. In patients with abnormalities of neuromuscular transmission however, the CMAP amplitude can vary with repetitive stimulation. In pre-synaptic disorders involving defects of neurotransmitter release, the baseline CMAP amplitude is characteristically small, with high-frequency nerve stimulation causing an incremental CMAP response as a result of calcium build-up in the pre-motor terminal. In contrast, in post-synaptic disorders the slight reduction in the release of the neurotransmitter acetylcholine (ACh) caused by low-frequency stimulation can cause the end-plate potential to fall below the threshold required to generate a muscle action potential in a proportion of the muscle fibres. This results in decrement of the CMAP amplitude. However, in many cases, whether the disorder is pre- or post-synaptic cannot be effectively differentiated using neurophysiological testing alone.

In CMS due to mutations in the gene encoding the pre-synaptic choline acetyltransferase (*CHAT*), the deficit is not in calcium-triggered ACh release, but in re-synthesis of ACh following re-uptake by the nerve terminal [2, 3]. Since the terminal contains a large reserve pool of ACh vesicles, a decremental response on RNS is only apparent once this pool has been exhausted. Hence, low-frequency stimulation typically produces no decrement, with the abnormality only becoming apparent following prolonged high-frequency nerve stimulation (e.g. 10 Hz for 5 min).

SFEMG determines the variability in the latency of neuromuscular transmission for individual muscle fibres within the same motor unit, or “jitter”. Increased jitter is more sensitive for detecting impaired neuromuscular transmission than RNS, but is less specific since the findings are essentially identical for both pre- and post-synaptic disorders and hence must be interpreted along with clinical symptoms and signs.

CMS may be particularly difficult to diagnose in neonates, in whom non-specific features such as generalised hypotonia, arthrogryposis and poor suck or cry may be the only clinical signs. In this age group, certain subsets of CMS may be associated with life-threatening episodic apnoea (CMS-EA); a rare, but potentially treatable cause of apparent life-threatening events (ALTEs). Whilst CMS-EA was initially described in association with mutations in *CHAT* [2], the genetic basis has expanded, and several more recently described CMS genes have been shown to be associated with EA (Table 1). Genetic diagnosis for these conditions is all the more imperative given that effective treatments may prevent these life-threatening crises, but treatment response varies depending on the genetic subtype. The underlying mechanism of EA in disorders of neuromuscular transmission is unknown. We reviewed the clinical course of 19 patients with CMS-EA, to demonstrate potential diagnostic pitfalls and to assess long-term prognosis.

**Table 1:** CMS genes associated with EA

Gene	Protein and function	Clinical features
*CHAT*	Choline acetyltransferase (ChAT); re-synthesis of acetylcholine (ACh) from choline and acetyl-CoA in pre-synaptic nerve terminal [2]	Can exhibit striking clinical variability both between and within families. Positive response to AChEIs is seen in almost all cases [3, 22]
*RAPSN*	Rapsyn; post-synaptic scaffolding protein, interacts with AChRs to induce clustering [23]	Two main phenotypes: late-onset with fatigable limb weakness, early onset characterised by arthrogryposis, high-arched palate, and facial, cervical and bulbar weakness [24–26]
*CHRNE* (fast channel)	Epsilon subunit of AChR, altered kinetic properties following binding of ACh to receptor [27, 28]	Severe weakness with crises [29]
*SLC5A7*	High-affinity choline transporter 1, resynthesises ACh in the pre-synaptic nerve terminal [30]	Phenotypes range from severe form with arthrogryposis, hypotonia and early lethality, to neonatal onset CMS with prominent EA [20]
*SLC18A3*	Vesicular acetylcholine transporter (VAChT), uptake of ACh into pre-synaptic vesicles [20]	Ptosis, ophthalmoplegia and fatigability. Deterioration of symptoms in cold temperature described in one case [20]
*COLQ*	ColQ, collagenic tail of AChE which anchors AChE in the post-synaptic membrane [31, 32]	Broad phenotype, from adult onset limb girdle CMS to early onset severe and progressive forms. Slowing of the pupillary light reflex (25% of cases). Worsening with AChEI therapy [31, 33]
*CHRND*	Delta subunit of AChR	Phenotype overlapping with rapsyn-CMS [34–36]
*SCN4A*	Voltage gated sodium channel (Nav1.4), influx of sodium ions into post-synaptic membrane and generation of muscle action potential [37, 38]	Relatively severe limb weakness, ophthalmoplegia and ptosis. Apnoeic attacks which persisted from infancy into early adulthood are described [38]
*MYO9A*	Unconventional myosin Myo9A, presumed pre-synaptic function [5]	Identified in two families, neonatal onset EA which responded dramatically to pyridostigmine and 3,4-DAP is described [5]

## Methods

Clinical information was reviewed from 34 patients who currently attend the CMS clinic at the John Walton Muscular Dystrophy Research Centre, Newcastle Upon Tyne, United Kingdom, and 929 patients from around the world who were referred to our laboratory for genetic testing between 1997 and 2011 in the context of a clinical suspicion of CMS following review by experienced neuromuscular clinicians [4]. Patients had convincing evidence of CMS on the basis of clinical features, neurophysiological studies, laboratory investigations (including measurement of AChR antibodies and serum creatine kinase) and, in many cases, a prior muscle biopsy. EA was noted as being present where the clinician had recorded recurrent periods of respiratory arrest during the disease course; patients described as having respiratory insufficiency (causing inadequate oxygenation) but lacking clear description of recurrent apnoeic events (with cessation of respiration) were not included. CMS-EA was clearly described in 32 patients; however, detailed clinical information and follow-up were available for 19 CMS-EA patients. For these cases, clinical, genetic and neurophysiological data were retrospectively reviewed. Genomic DNA samples or EDTA blood were provided to our laboratory from neuromuscular and neurology centres worldwide. Sanger-based mutation screening was carried out on a gene-after-gene basis according to phenotype. In one case, which has been previously described, the causative gene (*MYO9A*) was identified following whole exome analysis [5]. Informed consent was obtained from all participants by local institutions. All genetically undiagnosed patients had been screened for mutations in *CHAT, RAPSN, CHRNE, COLQ, DOK7, CHRNA1, CHRND, CHRNB1, SLC5A7* and *SLC18A3*. Neurophysiological assessment was performed at local institutions according to protocols described previously [6, 7]. Decrement was defined as a decrease in CMAP amplitude of 10% or more between the first and fourth CMAPs.

## Results

**Fig. 2:**
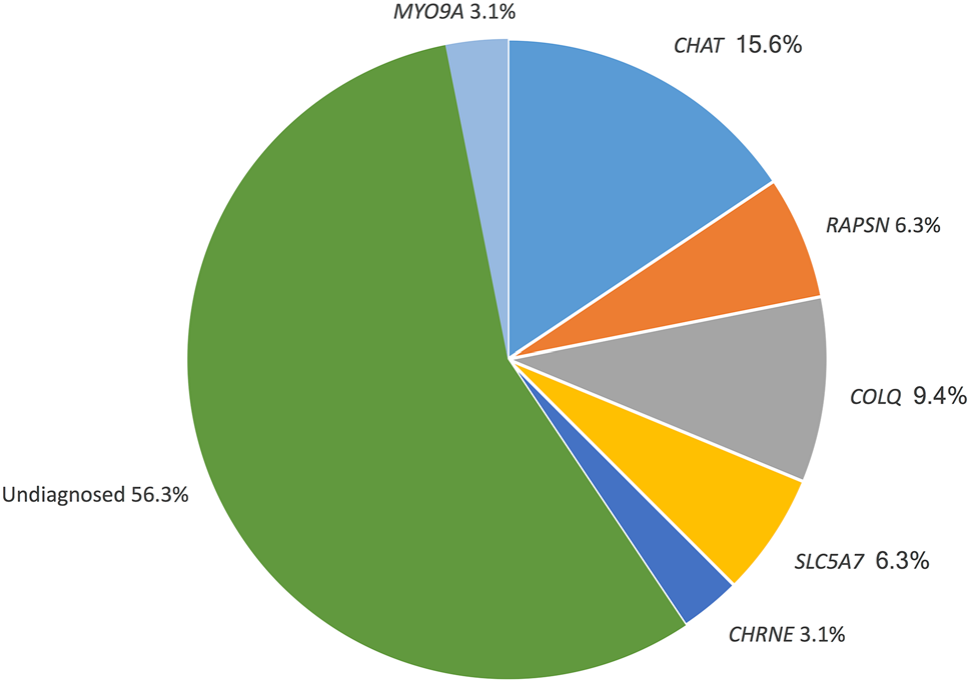
Proportion of CMS-EA subtypes in our patient cohort (*n*=32)

Episodic apnoea was described in 3% (*n* = 32/963) of CMS cases. Of the CMS-EA cases, 44% (*n* = 14/32) were genetically diagnosed, compared with 93% (*n* = 866/963) of all CMS cases. The most common causative mutations were in the *CHAT* gene (16% of CMS-EA cases), with mutations in *COLQ* (9%), *RAPSN* (6%), *SLC5A7* (6%), *MYO9A* (3%) and *CHRNE* (3%) being causative for the remainder of the cases with genetic diagnoses (Fig. 2).

In 13 cases, there was insufficient information on long-term follow-up and these were excluded from further analysis. Clinical features, neurophysiology and management are described, therefore, for the remaining 19 cases (Table 2).

**Table 2:** Clinical and neurophysiological features in 19 cases of CMS-EA

	Current Age, years	Causative gene; mutation	Cranial muscles	Weakness (distrib); Fatigability	Additional features	Cognition	Neurophysiology (age at assessment)	Treatment and response
Case 1	14	*CHAT;* 2081C > G (S694C) ex 18, 1061C > T (T354M) ex 10	Pto, bulb	Yes (prox); O/E		Severely impaired	RNS—no decrement on 10 Hz stimulation (5 months)	AChEI—transient improvement
Case 2	14	*CHAT;* 2081C > G (S694C) ex 18, 1061C > T (T354 M) ex 10	Pto	Yes (prox); O/E		Normal	RNS—no decrement on 3 Hz stimulation. **20% decrement on prolonged high-frequency stimulation of facial and distal muscles** (7 months)	AChEI—good response
Case 3	8	*CHAT;* 1408C > T (R470X) ex 11, 1730T > G (F580C) ex 13	Ophth, pto	Yes (glob); O/E	AMC	Mildly impaired	RNS—borderline decrement on 3 Hz stimulation, **90% decrement on prolonged high-frequency stimulation**. SFEMG - Increased jitter (3 months)	AChEI—good response. 3,4-DAP—good response
Case 4	3	*RAPSN*; 264C > A (N88 K) ex 2, 1169G > A (C390Y) ex 8	Pto, bulb	Yes (prox, WE, FE); Hx	AMC	Mildly impaired	RNS—20% decrement on 3 Hz stimulation (7 months)	AChEI—good response
Case 5	11	Unknown	Ophth, pto, bulb	Yes (prox, NE, FE); Hx		Normal	RNS—10% decrement on 3 Hz stimulation (10 months)	AChEI—no response. Salb—good response
Case 6	31	Unknown	Ophth, pto, bulb	Yes (glob); Hx		Mildly impaired	RNS—10% decrement on 3 Hz stimulation (11 years)	AChEI—good response. 3,4—DAP—no response
Case 7	16	Unknown	Ophth, pto, bulb	Yes (glob); No	AMC, hip dysplasia, scoliosis, high-arched palate	Mildly impaired	RNS—10% decrement on 3 Hz stimulation (2 years)	AChEI—no response. Salb—no response
Case 8	33	Unknown	No	Yes (dist); Hx		Normal	RNS—20–30% decrement on 3 Hz stimulation (3 years)	None tried
Case 9	14	*SLC5A7;* 331T > C (Y111H), 1252T > G (F418 V)	Ophth, pto, bulb	Yes (prox); O/E		Mildly impaired	RNS—no decrement on 3 Hz stimulation (3 years)	AChEI—no response. Salb—no response
Case 10	6	*SLC5A7;* 524A > G (Y175C), c.1030G > C (V344L)	Ophth, pto, bulb	Yes (glob); No		Severely impaired	RNS—70% decrement on 3 Hz stimulation (14 months)	AChEI—good response. Salb—no response
Case 11	1	Unknown	Ophth, pto, bulb	Yes (glob); Hx		Normal	RNS—no decrement on 3 Hz stimulation (5 months)	AChEI—no response
Case 12	7	Unknown	Pto, bulb	Yes (prox); Hx		Normal	RNS—no decrement on 3 Hz stimulation (5 years)	AChEI—good response. Salb—transient improvement
Case 13	13	Unknown	Ophth, pto, bulb	Yes (prox); Hx	AMC, hip dysplasia, high-arched palate	Normal	RNS—no decrement on 3 Hz stimulation (4 years). Repeat RNS—**significant increment in APB (25**–**34%) and ADM (29**–**41%) at 30** **Hz prolonged high-frequency stimulation** (7 years)	AChEI—good response
Case 14	1	Unknown	Pto, bulb	Yes (prox); Hx		Normal	RNS—decrement at 0.5 Hz stimulation, SFEMG—marked jitter and blocking (1 year)	AChEI—good response
Case 15	6	Unknown	Pto, bulb	Yes (prox); O/E		Normal	RNS—no decrement on 3 Hz stimulation, **prolonged high-frequency stimulation revealed decrement of 50% with partial recovery** (10 weeks)	AChEI—good response
Case 16	9	Unknown	Pto, bulb	Yes (prox); O/E		Normal	RNS—no decrement at 3 Hz, **71**–**84% decrement on prolonged high-frequency stimulation** with partial recovery (5 years)	AChEI—good response
Case 17	6	*CHRNE;* 43T > C (Y15H) ex 2, 113C > A (T38 K) ex 2	Ophth, pto, bulb	Yes (prox); O/E		Normal	RNS—decrement at 3 Hz in ADM, APB and ADM (8 months)	AChEI—transient improvement. Salb—good response.
Case 18	12	*CHAT;* 1642C > T (R548X) ex 15; 1669G > A (A557T) ex 15	Ophth, Pto, bulb	Yes (prox, NE); O/E		Mildly impaired	RNS—decrement at 3 Hz stimulation. SFEMG—borderline jitter (50 μs MCD) in deltoid (1 year)	AChEI—good response. Salb—good response.
Case 19	4	Unknown	Ophth, pto, bulb	Yes (axial); O/E	High-arched palate	Mildly impaired	RNS—no decrement at 3 Hz stimulation, **38% decrement on prolonged stimulation** (5 months). Repeat RNS—no decrement at 3 Hz, **48% decrement on prolonged stimulation**; SFEMG—jitter and blocking (4 years)	AChEI—good response

All genetically undiagnosed patients were screened for mutations in *CHAT*, *RAPSN*, *CHRNE*, *COLQ*, *DOK7*, *CHRNA1*, *CHRND*, *CHRNB1*, *SLC5A7* and *SLC18A3*

*3,4-DAP* 3,4-diaminopyridine, *AChEI* acetylcholinesterase inhibitor, *AMC* arthrogryposis multiplex congenital, *bulb* bulbar weakness, *dist* distal, *FE* finger extensor weakness, *glob* global, *Hx* fatigability reported on clinical history, *NE* neck extensor weakness, *O/E* fatigable on examination, *ophth* ophthalmoplegia, *prox* proximal, *pto* ptosis, *RNS* repetitive nerve stimulation, *Salb* salbutamol, *SFEMG* single-fibre electromyography

The cases which demonstrated decrement on RNS only after prolonged high frequency stimulation are highlighted in bold

### Clinical features

All cases were proband, apart from cases 14 and 15 who are siblings. There was no family history of a neuromuscular disorder in any case, and consanguinity was reported in only two families. The disease manifested at birth in 84% (*n* = 16) of cases, with the remaining three cases presenting within the first 2 months of life. Signs present at birth were hypotonia (16 cases) and arthrogryposis (4 cases). Antenatal complications were recognised in 42% (*n* = 8) of patients, and included reduced foetal movements and/or polyhydramnios.

Age of onset of EA was variable. Most patients had their first apnoea in the hours following birth (*n* = 5, 26%) or in the first 4 weeks of life (*n* = 5, 26%). For four cases (21%), the first apnoeic event occurred between the age of 1–4 months, and four cases developed EA between 4 and 12 months of age. In one case, EA did not occur until the age of 18 months.

Cognitive development was abnormal in nine cases (47%); in three cases, this was severe and considered to be secondary to hypoxic brain damage. Brain MRI was available for ten patients, and described as normal in seven, with two cases showing features in-keeping with hypoxic ischaemic encephalopathy, and one case (with *CHAT*-CMS) showing hypoplasia of the cerebellar vermis.

Median diagnostic delay from first symptoms to the clinical diagnosis of CMS (prior to genetic confirmation) was 8 months (range 1–96 months). Differential diagnoses included laryngomalacia, epilepsy, cardiac conduction defects, congenital hypothyroidism, Prader–Willi syndrome and spinal muscular atrophy.

### Apnoeic events and respiratory function

Infections, including respiratory tract infections, were the most common precipitating factor for apnoeas, reported in 74% (*n* = 14) of patients (Table 3). Apnoeas were also reported during feeding, stress, crying, increased activity and increased environmental temperature. Frequency of apnoeas varied significantly, independent of genotype, from approximately one episode per month, to very frequent episodes occurring up to 40 times per day.

**Table 3:** Apnoeic events and respiratory function in our patient cohort

	Precipitating events	Associated symptoms during apnoea	Recurrent LRTI	Respiratory function between episodes
Case 1	Feeding, infection, stress	Hypotonia, bulbar weakness, ptosis	Yes	Normal
Case 2	Feeding, infection, stress	Hypotonia	Previously, now resolved	Normal
Case 3	Infection	Hypotonia	Previously, now resolved	Normal
Case 4	Infection, feeding	Hypotonia	Yes	Normal
Case 5	Infection, during sleep	Hypotonia, bulbar weakness	Yes	Normal
Case 6	Stress, infection, higher temperature	Ptosis, bulbar weakness, hypotonia	Previously, now resolved	Normal
Case 7	Crying	Hypotonia	No	Normal
Case 8	Infection	None noted	Previously, now resolved	Normal
Case 9	Feeding, infection	Ptosis, bulbar weakness, hypotonia	No	Normal
Case 10	Infection	Hypotonia	Yes	Permanently ventilated
Case 11	Infection, feeding, crying	None noted	Yes	Normal
Case 12	Feeding, infection	Hypotonia, facial weakness	Yes	Impaired
Case 13	Feeding	No	Yes	Normal
Case 14	Feeding	Hypotonia, ptosis, cyanosis	Yes	Not assessed
Case 15	Infection, increased activity, sleep	Hypotonia	Yes	Normal
Case 16	Infection, increased activity, sleep	Hypotonia, bulbar weakness	Yes	Normal
Case 17	Feeding	Hypotonia	No	Normal
Case 18	Feeding, infection, during sleep	Hypotonia, cyanosis	Yes	Impaired
Case 19	Infection, feeding	Hypotonia, cyanosis	Yes	Impaired

*LRTI* lower respiratory tract infection

During apnoeic episodes, myasthenic features typically worsened, with worsening of hypotonia, bulbar weakness and ptosis reported in 14, 6 and 4 cases, respectively. Approximate duration of events ranged from 30 s to over 30 min (persisting until the patient was intubated and ventilated, and resulting in permanent brain damage), but mean duration of a typical event was 2 min.

**Fig. 3:**
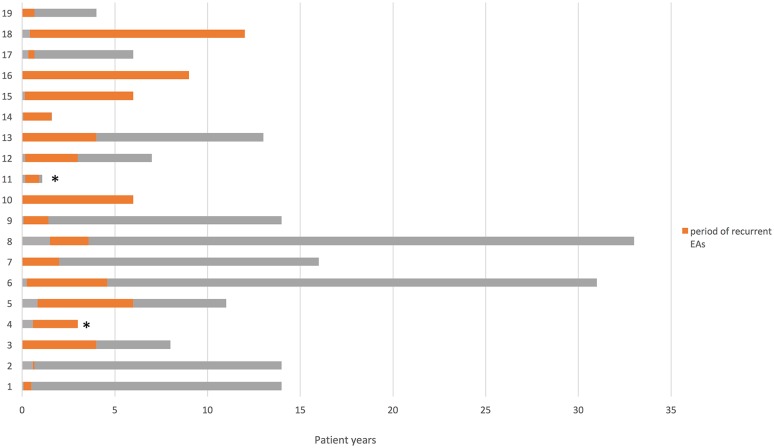
Resolution of apnoeic events over time: For the majority of cases, the period of recurrent EAs (orange) during a patient’s life span (gray) began in the first months of life and resolved in early childhood. Cases marked * are deceased

There was significant morbidity and mortality in the cohort, with two patients having died following prolonged apnoeas, aged 11 months (genetically undiagnosed) and 3 years (with *RAPSN* mutations), respectively, and a further case with severe hypoxic brain injury being permanently ventilated (*SLC5A7* mutations). However, overall progressive improvement and tendency towards resolution of apnoeic episodes was reported in the majority of cases (Fig. 3). For 11 cases there was complete remission of apnoeas (mean age of resolution 2 and 5 months). A further five cases showed tendency towards remission but still experienced apnoeas during infections. There was no genotype–phenotype correlation between cases who had remission of EA and those who still experienced apnoeas.

### Neurophysiology

All patients had neurophysiological assessment; 4 cases had RNS and SFEMG, and 15 cases had been investigated using RNS alone. Abnormal RNS was seen in 15/19 cases (79%); in four cases, in whom only RNS was performed, no decrement was detected. In 5 of the 15 cases with abnormal RNS, stimulation at 3 Hz was borderline or negative, but prolonged high-frequency RNS (10 Hz for 5 min) revealed marked decrement. In a further patient (genetically undiagnosed) increment was detected on prolonged stimulation. All cases who had SFEMG in addition to RNS demonstrated increased jitter.

### Management

18 cases had been treated with acetylcholinesterase inhibitors (AChEIs) at some point. Of these, 67% (*n* = 12) had a sustained clinical improvement, which included all patients with mutations in *RAPSN* and *CHAT*, one patient with *SLC5A7* mutations and seven patients who were genetically undiagnosed. Transient improvement but subsequent deterioration following commencement of AChEIs was seen in two patients (both genetically undiagnosed). AChEIs had no effect in four cases (three genetically undiagnosed, and one *SLC5A7*-CMS). The β_2_-agonist salbutamol resulted in clinical improvement in three cases (genetically undiagnosed, fast channel syndrome and *CHAT*-CMS), transient improvement in one case (undiagnosed) and no response in the remaining three cases (one undiagnosed, and two with *SLC5A7*-CMS). 3,4-Diaminopyridine (3,4-DAP) was used as an adjunctive therapy in two cases, resulting in improvement in one (with *CHAT*-CMS) and no response in the other (genetically undiagnosed). All six cases in whom abnormal RNS had only been detected following prolonged high-frequency stimulation had clinical benefit from AChEI therapy.

Most patients (*n* = 14, 74%) had normal respiratory function between apnoeic events. Three cases required nocturnal non-invasive ventilation (one with *CHAT*-CMS and two genetically undiagnosed cases), and one was permanently ventilated via tracheostomy. Hospital attendances were common, with 41% (*n* = 8) of cases requiring frequent (> 1 per year) admissions. Fourteen patients had required ventilation during periods of respiratory crisis (11 invasive and 3 non-invasive ventilation).

## Discussion

Apparent life-threatening events (ALTEs), defined as episodes characterised by some combination of apnoea, skin colour change, change in muscle tone and choking or gagging [8], have an extremely broad differential diagnosis. Causes include epilepsy, cardiac arrhythmia or structural cardiac defects, gastro-oesophageal reflux disease, respiratory tract infections and upper airway obstruction [9–12]. ALTEs are most common in the first 10 weeks of life, with cyanosis and apnoea often being the most prominent or only symptoms [11]. CMS is rarely considered as a possible differential diagnosis for ALTEs, thus many patients undergo extensive investigation before the possibility is considered. In our cohort, average diagnostic delay was substantial at 8 months. Worsening of myasthenic symptoms during apnoeas was frequently observed, and these features may provide a diagnostic clue. In practice, however, many patients may be admitted directly to intensive care units and may not be examined by a paediatric neurologist or neuromuscular specialist during these crises.

In early infancy, abnormal neurophysiology tests may be the only indication of a NMJ disorder. However, we observed in this cohort that without specific neurophysiological assessment, a pre-synaptic abnormality may go overlooked. In 32% (*n* = 6) of cases, no abnormality was observed at low-frequency RNS, with decrement or increment only becoming apparent following prolonged high-frequency stimulation. In two of these cases, the genetic diagnosis was of ChAT deficiency, in which re-synthesis of ACh is known to be impaired. In the other four cases, the genetic diagnosis was unknown, but it may be that these also affect proteins involved in this pathway. Failure to perform this additional test could result in the diagnosis of CMS-EA being discounted. Furthermore, all of these cases showed a positive response to AChEI therapy. Such prolonged studies can be uncomfortable for the patient, and may require sedation. Nevertheless, given the risk of fatal apnoeas if the diagnosis is missed, and the potential for clinical improvement with AChEIs, these prolonged studies should be performed if deficiency of ACh re-synthesis is suspected.

The underlying mechanism(s) giving rise to these sudden and recurrent apnoeas is yet to be characterised. Respiratory control mechanisms respond to input from neural and chemical receptors, which are integrated by the respiratory centres in the medulla and pons. These subsequently provide neuronal drive to respiratory muscles, maintaining upper airway patency and determining the level of ventilation. Recurrent apnoea could be due to an abnormality at any point along this axis. The majority of mutations causing CMS-EA lead to pre-synaptic defects (Fig. 1), which have important functions for both central and neuromuscular synaptic function. This, along with the tendency for patients to have normal respiratory function between apnoeas, could point to a centrally mediated mechanism. In addition, the proportion of patients with impaired cognitive function in our cohort (47%) was higher than expected and is not something that has been previously well-characterised in disorders of the NMJ. Any impairment of higher function in CMS-EA patients is often explained by hypoxic brain damage due to repeated respiratory failure, although the possibility of impaired CNS maturation due to reduced neurotransmitter in central cholinergic neurons has not been fully explored. The most common CMS-EA gene, *CHAT*, has important functions in both central and peripheral synapses, and deficiency of ChAT has been reported in Alzheimer’s disease, idiopathic Parkinson’s disease, Huntington’s disease and schizophrenia [13–16]. Furthermore, in cases of sudden infant death syndrome, decreased activity of ChAT has been demonstrated in the CNS [17, 18]. Cognitive and behavioural have also been described in the recently identified CMS subtype due to mutations in *SLC5A7*, which encodes the high-affinity choline transporter 1, necessary for uptake of choline from the synaptic space for the synthesis of ACh at central and peripheral cholinergic synapses [19]. A further CMS-EA gene involved in ACh release, *SLC18A3*, encodes the vesicular acetylcholine transporter (VAChT), which mediates the vesicular storage of ACh in the central and peripheral nervous system. *SLC18A3* mutations are associated with a severe CMS phenotype and learning difficulties are also described [20, 21]. The CMS-EA genes *SNAP25B*, *VAMP1* and *MUNC13* encode proteins of the SNARE complex. These are essential for calcium-triggered exocytosis at central and neuromuscular synapses; therefore, it is unsurprising that CMS due to mutations in these genes may be associated with epilepsy and impaired brain development [22, 23]. Abnormalities of the CNS have also been reported in CMS due to mutations in the unconventional myosin gene *MYO9A*. MYO9A also appears to have pre-synaptic function and localization at the NMJ, although the mechanism for NMJ dysfunction in these patients is yet to be determined [5].

Administration of one or more drugs is indicated once the diagnosis of CMS-EA is established. Several drugs have shown convincingly positive effects in reducing the frequency of apnoeas, including AChEIs, 3,4-DAP and sympathomimetics (ephedrine or salbutamol). Because of the sudden nature of these attacks, parents should be provided with an inflatable rescue bag and fitted mask, trained in CPR and may be provided with a home apnoea monitor. Parents may also be instructed to give additional AChEI doses during infection or other potential precipitating events.

We recognise that this study has several limitations. We include a small number of patients, contributed to by the rarity of the condition. Data were collected retrospectively and over a long period of time (1997–2011) during which the awareness of CMS and availability for genetic testing has increased. However, even in this patient cohort, who continues to be tested for new causative CMS genes as they are discovered, a relatively large proportion (56%) of cases remain genetically undiagnosed. CMS-EA is a challenging diagnosis, with several mimics, and this proportion could suggest an alternative diagnosis other than a neuromuscular transmission defect in these patients. However, it may also reflect further CMS causing genes which have not yet been discovered.

Given the likelihood of recurring episodes, the potential for psychomotor impairment due to secondary hypoxic brain damage, and the positive effect of available treatments, CMS-EA is an important diagnosis not to miss. Prompt diagnosis and initiation of treatment and ventilatory support is imperative in early life, when EA may be frequent, but the majority of cases will exhibit a tendency towards remission. Improved understanding of the mechanism of this condition may provide insights into further causative genes for genetically undiagnosed patients, and lead to improved diagnostic and treatment strategies.
